# *Obuntu bulamu*: Parental peer-to-peer support for inclusion of children with disabilities in Central Uganda

**DOI:** 10.4102/ajod.v12i0.948

**Published:** 2023-01-30

**Authors:** Ruth Nalugya, Harriet Nambejja, Claire Nimusiima, Elizabeth S. Kawesa, Geert van Hove, Janet Seeley, Femke Bannink Mbazzi

**Affiliations:** 1 MRC/UVRI & LSHTM Uganda Research Unit, Kampala, Uganda; 2 Spina Bifida and Hydrocephalus Associations of Uganda, Kampala, Uganda; 3 Faculty of Psychology and Educational Sciences, Ghent University, Ghent, Belgium; 4 London School of Hygiene & Tropical Medicine, London, United Kingdom

**Keywords:** inclusion, participation, inclusive education, peer support, belonging, Ubuntu, obuntu bulamu

## Abstract

**Background:**

*Obuntu bulamu*, a peer-to-peer support intervention for children, parents and teachers to improve the participation and inclusion of children with disabilities (CwD), was developed and tested in Uganda. The intervention consisted of disability-inclusive peer-to-peer training and support activities. In this article, parent participation in and evaluation of the intervention are discussed.

**Objectives:**

The study aims to evaluate the acceptability and feasibility of the intervention.

**Methods:**

A qualitative Afrocentric intervention study was implemented in 10 schools in Wakiso district in Central Uganda. Researchers purposely selected CwD aged 8–14 years, their peers and parents from 10 primary schools with on average three CwD per school. A total of 64 study parents (33 parents of CwD and 31 peers) were interviewed at baseline and endline. Two focus group discussions were held with 14 parents at midline. Parents also participated in a consultative meeting about the intervention design at baseline and two evaluation and feedback workshops at midline and endline. Thematic data analysis was conducted.

**Results:**

Findings showed that parents found the intervention inspiring, acceptable, culturally appropriate and supportive, as it built on values and practices from their own cultural tradition. Parents reported that the intervention enhanced a sense of togetherness and belonging and helped them to develop more positive attitudes towards CwD and disability inclusion. They felt the intervention increased participation and inclusion of CwD at home, school and in communities.

**Conclusion:**

The *Obuntu bulamu* peer-to-peer support intervention is an acceptable, culturally appropriate intervention with the potential to improve inclusion of CwD. Further studies are recommended to measure the effectiveness of the intervention.

**Contribution:**

The paper contributes to existing evidence that there is need for more Afrocentric interventions, which built on cultural values and practices. Interventions based on indigenous values have a greater potential to be acceptable, can foster integration and are likely to be more sustainability to achieve disability inclusion. In the article we describe parental perspectives of the Obuntu bulamu intervention, an intervention to improve inclusion of children with disabilities, which was designed by children, parents, teachers, educationalists, and academics from Uganda.

## Introduction

The Convention on the Rights of the Child (CRC) states that the family has the key responsibility to ensure the fundamental rights of children. The family is the primary setting within which children are cared for and parented; it is where the first significant relationships develop and the foundations of children’s development take place (Carter & Boezaart 2016; Lawson 2006; United Nations Children’s Fund 1989). Degbey and Saee (eds. 2012) observed that the extended family can meet the emotional needs of all involved as a cohesive unit, which ideally provides economic, social and psychological security to all its members. Adinlofu (2009) mentioned that the family ensures procreation of children and provides for the early care and training of children.

Recognising the critical role that the family plays in the inclusion and provision of care for children living with complex disabilities has resulted in shifting from focusing on the child to considering the needs of the whole family (Rosenbaum et al. 1998). According to Adinlofu (2009), performing the responsibilities of raising and relating to children in such a manner that the child is well prepared to realise his or her full potential as a human being requires interpersonal skills, which make emotional demands.

Several interventions, such as parenting and stress management interventions and inclusive education, have been piloted to promote the inclusion of children with disabilities (CwD) across a variety of settings (Simplican et al. 2015). Given the difficulties faced by parents of CwD, a range of approaches and programmes have focused on supporting parents with parenting skills and engagement, for example, programmes covering interactional, instructional and family systems, as well as positive behavioural support (Breiner, Ford & Gadsden 2016). These programmes include training in supporting parents with knowledge, attitudes and practices that promote the children’s physical and mental development and prevent the occurrence of emotional and behavioural problems, youth crime, risky behaviour, exploitation, discrimination and violence against other girls and boys (Choudhury & Jabeen 2008; Roper 2014; Shenderovich et al. 2018; Siu et al. 2017). Parental involvement for children with CwD is crucial because parents have a unique understanding of their child’s needs and therefore are regarded as the best advocates in asserting their children’s rights and making decisions for them (Ceka & Murati 2016; Öztürk 2017). In addition, it has been argued that if parents are deeply involved in the inclusion processes of their children, their worries about their children’s futures will decrease (Mafa & Makuba 2013).

Although there have been notable and creative changes within the global disability-inclusive development, there is growing recognition that the approaches adopted to achieve the goals of universal access and quality education and family-centred interventions to increase parent involvement in disability inclusion are inadequate (Goldman & Burke 2017; United Nations Children’s Fund 1989; World Economic Forum 2015). Issues highlighted within the adopted approaches include the failure to engage parents and local communities in supporting education, embracing a holistic approach to disability inclusion, acknowledging the complexity of the barriers impeding children’s access to school and listening to the concerns expressed by children themselves concerning their education. There is also a failure to build a culture of education in which all children are equally respected and valued that addresses children’s rights to act whilst participating and living in a learning arena or to ensure schools are vibrant centres for community action and social development (Green 2007; Kamenopoulou 2018; Stofile 2008).

Mitra and Shakespeare (2019) argued that there is a need to reconsider selecting approaches that are relevant to aspects of life and replace and/or supplement activities and participation with a more holistic concept. Indigenous concepts have the potential to promote acceptance (Bannink Mbazzi et al. 2020) and active participation and uncover the positive and the ambivalent views of disability and assistance (Miles 2003).

An indigenous peer-to-peer support intervention for inclusion (*obuntu bulamu*) was piloted to evaluate if the intervention is acceptable and can potentially improve attitudes of peers and teachers towards CwD in school, participation of CwD at home and school and the quality of life of CwD. The intervention consisted of a peer-to-peer training package over two school years and involved parents, teachers and children. In this article, findings are described regarding the participation by parents in, and their evaluation of, the peer-to-peer support component within the *obuntu bulamu* intervention.

## *Obuntu bulamu*: An African intervention model

*Obuntu bulamu* is a Luganda term for an accepted and consistent behaviour that signifies a shared set of values, which promote well-being, togetherness and unity. It is closely linked to the Ubuntu philosophy (I am because we are), which has been described as a key component of African disability discourse (Berghs 2017; Chataika & McKenzie 2013; Mugumbate & Nyanguru 2013). The *obuntu bulamu* framework, which is based on this concept, starts with recognition and belonging. The emphasis is upon the importance of belonging (the attachment to people and places in a person’s life) before being (who the person is) and becoming (things the person does through life) can take place, which is explained elsewhere (Bannink Mbazzi, Nalugya & Van Hove 2019). The *obuntu bulamu* study explores African concepts of disability and inclusion with an emphasis on belonging and family and community responsibilities. The intervention was developed and tested with CwD, parents, teachers, academics, health and rehabilitation workers and community and district leaders in Uganda (Bannink Mbazzi et al. 2020). The intervention promotes social responsibility, use of culturally appropriate methods and locally available curricula and materials to achieve change. The overall study hypotheses are that the intervention will result in the following outcomes:

1. Improve inclusion and participation in school, resulting in increased education access, retention and learning outcomes of CwD, classroom and playtime interaction of CwD, their peers and teachers, as well as inclusive teaching methods and attitudes used by teachers.
2. Increase inclusion in the home, resulting in increased participation in daily living activities and home interactions between household members and CwD.
3. Improve inclusion in community activities, leading to improved attitudes of community members towards CwD and increased participation of CwD in community activities.
4. Improve participation of CwD and their families in research, including in data collection, interpretation of findings and dissemination of results.
5. Improve quality of life for CwD.

**FIGURE 1:**
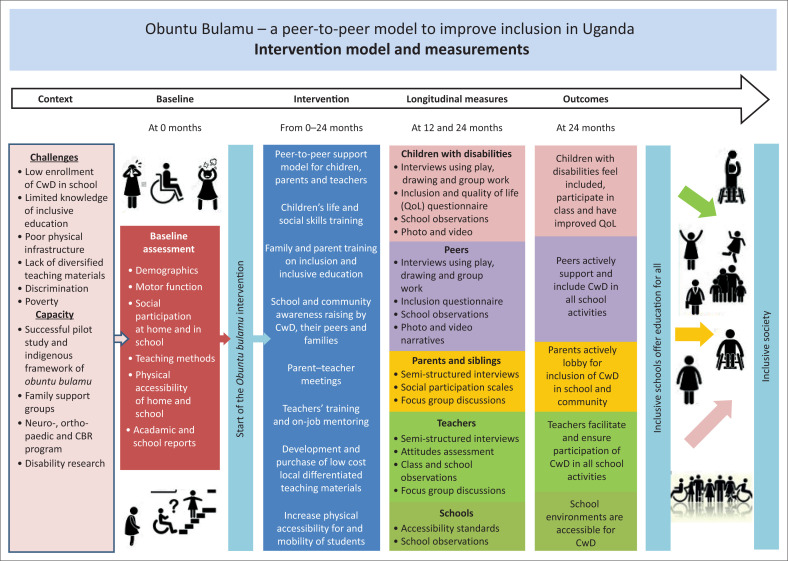
Theory of change for the *obuntu bulamu* intervention.

In this article, qualitative study findings will be discussed for outcomes (2), (3) and (4) from the parents’ perspectives. The theory of change (Figure 1) highlights the pathway from the context, the intervention and the measures to outcomes and impact.

Figure 1 shows the study’s proposed intervention pathway and components that were expected to cause change. It outlines the different intervention packages and levels and the specific changes that were expected because of the study being implemented. The arrows show causal pathways that were likely to determine the direction of the relationship between these changes and how they lead to the long-term outcomes and impact to which the intervention is intended to contribute.

## Parents’ involvement in the *obuntu bulamu* intervention

Parents of CwD and their peers were involved in the peer support intervention through a community disability group to enable support at the family, school and community levels. They were engaged throughout the intervention cycle through a baseline consultative meeting and interactive evaluation workshops at midline and endline. In the interactive workshops, 160 study participants (parents of CwD, parents of CwD’s peers, CwD and their peers, teachers) and representatives from Kyambogo University and the Ministry of Education and Sports (MoES) and District Education Office took part. In November 2019, preliminary study findings were disseminated at a stakeholders’ workshop and parents provided input into their component for future parent peer-to-peer intervention.

The intervention the parents received consisted of three training sessions for parents and quarterly support activities delivered by ‘focal parents’ over a period of two years. The focal parents had participated in a pilot study in which they had received training on inclusive education practices and had expressed an interest in participating in future studies. The ‘focal’ or ‘peer’ parent had multiple roles: he or she provided a listening ear and emotional support, shared information and assisted a parent to raise awareness and address other issues linked to disability inclusion, such as poverty and sometimes marital issues. Focal parents visited parents at least once in a school term over a period of two years. In case of difficult circumstances or absenteeism of the child at school, the focal parent conducted additional visits.

The three disability inclusion group training sessions that parents received focused on togetherness and belonging. The group training was conducted by Ugandan disability inclusion experts. The training was organised on a termly basis over a one year school period. The training was a 1-day participatory group session which aimed to promote critical thinking, problem solving and peer support in relation to disability inclusion. The training sessions focused on inclusive education (the meaning of inclusive education in Uganda, disability models and trends, rights associated nationally and internationally with disability and reflections on the challenges faced in raising a child with a disability), the role of parents in the learning process (supporting the child’s learning at school and home and parent–teacher meetings) and supporting parents in raising awareness in their communities and school to promote inclusive education (through community activities and visits to schools and homes). The training included parents of CwD and parents of the peer children who had been selected to provide peer support to CwD.

## Methodology

### Study setting, design and participants

This qualitative Afrocentric intervention study was implemented in 10 communities in the Wakiso district in Central Uganda. The overall study used both culturally adapted ‘international standards and tools’ (also described as an adaptive evaluation approach by Carden and Alkin (2012) and Chilisa and Major (2015) and more qualitative and ‘Afrocentric’ methods (Mkabela 2005). The study was conducted between 2018 and 2019 in Wakiso District, Uganda. A total of 64 parents (33 parents of CwD and 31 peer parents of children without disabilities) were recruited to participate in the study: 33 families with a CwD from 10 mainstream primary schools were purposively selected based on existing data about the child’s age, school class, impairment and impairment effects, and the 31 peer parents were identified through the peers selected by CwD. After CwD were selected, each one of them was asked to select a peer from their class. This process was guided by the child, with suggestions from the class teacher and parents. Peers were often playmates, assistants or caretakers in the current class setting who had shown an interest in the CwD. After peers expressed an interest, parents of each peer were contacted. All the participants received information about the study and parents or caregivers signed written consent forms, whilst children with the cognitive capacity who agreed to take part assented.

### Data collection

The *obuntu bulamu* study collected quantitative and qualitative data from children, parents and teachers. This article describes the findings that came from the data collected from in-depth interviews and focus group discussions (FGDs), as well as training and consultative meetings and workshops, as shown in Table 1.

**TABLE 1:** Data collection tools, timelines and purpose.

Data collection tool	Target group or participants	Number	Intervention phase	Information collected
Key informant interviews (KII)	Parents of CwD and peers	126	64 at baseline and 62 endline	Demographic data about the family, impairment-related information (from a medical and rehabilitative perspective), school attendance and social and general well-being collected at baseline, disability, inclusion, daily life activities, healthcare, education and support and more specifically on the *obuntu bulamu* intervention at endline.
FGD	Parents of children with disabilities	2	Midline	Disability, inclusion, daily life activities, healthcare, education and support and more specifically on the *obuntu bulamu* intervention at midline.
Consultative meetings and validation workshops	Children, parents and teachers	2	Midline and endline	Parental views on inclusion and support needs to develop and test the intervention. Presentation of concepts and findings and discussions about the intervention with parents, children and teachers. Validation of findings and presentations of findings with parents at endline.

FGD, focus group discussions; CwD, children with disabilities.

Qualitative data were collected to assess perceptions and acceptability of the intervention on peer support under the parents’ component (see Table 1). This included baseline and endline key informant interviews (KIIs) amongst 124 caregivers and their peers (62 caregivers and 62 peers). Two midline-focused group discussions were conducted with caregivers of CwD (one with eight women and one with six men). Two consultative or validation workshops were held with 126 participants including children, parents, teachers, community-based rehabilitation workers, academicians, district and ministry officials.

The KIIs and FGDs were moderated by two female research assistants, trained and experienced in both quantitative and qualitative data collection in social science research studies in the region. Both are Ugandan nationals fluent in Luganda, the language spoken in the Central Region of Uganda. During the FGD they were aided by a note-taker. Quality checks were conducted by the investigators, but none of the investigators participated in FGDs or KIIs in order to prevent influencing responses by their presence.

The KIIs were conducted in Luganda at the parents’ homes or another place of choice. The FGDs were held on weekends in a meeting room in the area where parents resided. On average, KIIs lasted 45 min whilst FGDs lasted 90 min. All KIIs and FGDs were audio-recorded, transcribed verbatim, translated in English and back translated by native Luganda speakers with a good education in English. Written informed consent was obtained from all participants.

Training reports were written by the training facilitators, research team members and peer parents after every training session. The reports included the topics covered in the training and feedback and questions asked by parents, as well as recommendations. Consultative and validation workshop reports were written by the research team members after the meetings and included the preliminary study findings shared, feedback received and recommendations for the next phase of data collection or study.

### Data analysis

Analysis of KII and FGD data were managed using NVivo 10 (QSR International, Melbourne, Australia). Data were reviewed following a thematic approach using framework analysis, a matrix-based system for organising, reducing and synthesising data (Vogel et al. 2013). A codebook was developed by three study team members and imported into NVivo 12. The thematically organised data were reviewed and synthesised into meaningful themes, and quotes were selected to highlight, explain or describe relevant themes. Data saturation was discussed by the analysis team, and they were informed about the number of FGDs and KIIs conducted. The analysis provided an in-depth understanding of participants’ perception of the intervention, interactions with the intervention components, mechanisms of impact and how these affected intervention outcomes.

### Ethical considerations

Ethical approval for the study was obtained from the Uganda Virus Research Institute Research Ethics Committee (ref. no. GC/127/18/02/633) in Entebbe and the Ethics Committee of the Faculty of Psychology and Educational Sciences of Ghent University (Bannink 2017/6). Overall permission to conduct the research was obtained from the Uganda National Council for Science and Technology (ref. no. HS SS4557).

## Results

### Participant characteristics

The sociodemographic characteristics of the 64 parent participants are summarised in Table 2. Out of 33 parents with a child with a disability, four had more than one child with a disability. Amongst the peer parents, one had a child with multiple disabilities. A total of 89% of the parents were female. The parent’s relationship to the child were mostly as the mother 57 (89.06%), followed by 7 (10.94%) fathers.

**TABLE 2:** Demographic characteristics of the participants.

Variable	Description	Parents of CwD (*n* = 33)					Parents of peers (*n* = 31)					Total	
		*n*	%	Mean	SD	Range	*n*	%	Mean	SD	Range	*n*	%
Gender	Female	31	93.94	-	-	-	26	83.87	-	-	-	57	89.06
	Male	2	6.06	-	-	-	5	16.13	-	-	-	7	10.94
Age of parents	under 35 years	14	42.42	-	-	-	12	38.71	-	-	-	26	40.63
	35–50 years	16	48.48	-	-	-	13	41.94	-	-	-	29	45.31
	51 years and above	3	9.09	-	-	-	6	19.35	-	-	-	9	14.06
Age of child	-	-	-	10.5	2.05	08–15	-	-	9.34	1.84	07–14	-	-
Disability of child	Autism spectrum disorder	4	12.50	-	-	-	0	-	-	-	-	4	10.81
	Down syndrome	2	6.25	-	-	-	0	-	-	-	-	2	5.41
	Hearing impairment	4	12.50	-	-	-	1	20.00	-	-	-	5	13.51
	Hydrocephalus	4	12.50	-	-	-	0	-	-	-	-	4	10.81
	Intellectual disability	8	25.00	-	-	-	0	-	-	-	-	8	21.62
	Muscular dystrophy	2	6.25	-	-	-	0	-	-	-	-	2	5.41
	Physical disability	1	3.13	-	-	-	3	60.00	-	-	-	4	10.81
	Spina bifida	4	12.50	-	-	-	0	-	-	-	-	4	10.81
	Visual impairment	3	9.38	-	-	-	1	20.00	-	-	-	4	10.81
Marital status	Married	19	57.58	-	-	-	16	51.61	-	-	-	35	54.69
	Separated	1	30.30	-	-	-	7	22.58	-	-	-	17	26.56
	Single	1	3.03	-	-	-	5	16.13	-	-	-	6	9.38
	Widowed	3	9.09	-	-	-	3	9.68	-	-	-	6	9.38
Education level	University	2	6.06	-	-	-	3	9.68	-	-	-	5	7.81
	Postsecondary, vocational	4	12.12	-	-	-	3	9.68	-	-	-	7	10.94
	Secondary school	12	36.36	-	-	-	10	32.26	-	-	-	22	34.38
	Primary school	15	45.45	-	-	-	15	48.39	-	-	-	30	46.88
Average net income in UGX (monthly)	< 200 000	25	75.76	-	-	-	25	80.65	-	-	-	50	78.13
	200 000–400 000	6	18.18	-	-	-	1	3.23	-	-	-	7	10.94
	400 001–600 000	1	3.03	-	-	-	4	12.90	-	-	-	5	7.81
	> 600 000 UGX	1	3.03	-	-	-	1	3.23	-	-	-	2	3.13
House ownership	Owned	13	39.39	-	-	-	13	41.94	-	-	-	26	40.63
	Relatives	2	6.06	-	-	-	0	-	-	-	-	2	3.13
	Rented	18	54.55	-	-	-	18	58.06	-	-	-	36	56.25
All children going to school	Yes	22	66.67	-	-	-	15	48.39	-	-	-	37	57.81
	No	11	33.33	-	-	-	16	51.61	-	-	-	27	42.19
Primary way of moving child	Walking	27	81.82	-	-	-	31	100	-	-	-	58	90.63
	Using assistive devices	5	15.15	-	-	-	0	-	-	-	-	5	7.81
	Crawling	1	3.03	-	-	-	0	-	-	-	-	1	1.56
Child’s method of communication	Nonverbal gestures	4	12.12	-	-	-	1	3.23	-	-	-	5	7.81
	Verbal speech 1–2-word phrases	9	27.27	-	-	-	2	6.45	-	-	-	11	17.19
	Verbal speech, full sentences	20	60.61	-	-	-	28	90.32	-	-	-	48	75.00
Occupation	Homemaker	13	39.39	-	-	-	1	3.23	-	-	-	14	21.88
	Self-employed	12	36.36	-	-	-	14	45.16	-	-	-	26	40.63
	Unemployed	2	6.06	-	-	-	7	22.58	-	-	-	9	14.06
	Formal employment	6	18.18	-	-	-	9	29.03	-	-	-	15	23.44
Class index, child	Nursery	2	6.25	-	-	-	2	6.25	-	-	-	4	6.25
	Lower primary	19	59.38	-	-	-	20	62.50	-	-	-	39	60.94
	Upper primary	8	25.00	-	-	-	8	25.00	-	-	-	16	25.00
	Step-up class	3	9.38	-	-	-	2	6.25	-	-	-	5	7.81

CwD, children with disabilities.

The median parental age was 36 (SD = 11.83). The majority were married and had completed primary school.

The average household size was 4.8 (SD = 2.05, range 1–9) for parents with CwD and 5.3 (SD = 3.6, range 0–15) for peer parents. The average number of children per household was 4.1 (SD = 1.84, range 1–9) for parents of CwD and 4.0 (SD = 2.50, range 0–12) for peers’ parents. Children with a disability were more often in lower classes (usually for younger children), for example, in primary 2, 5 out of 10 (50.00%) CwD were aged 10, and 5 out of 11 (45.45%) peers were 8 years old. Out of the 7 parents who reported to earn between UGX 200 000 and 400 000 a month, 6 (85.71%) were parents of CwD and one (14.29%) was the parent of the peer counterpart. More parents of CwD, 4 (80.00%), earned between UGX 400 000 and 600 000 compared with the peer parents, where there was only 1 (20.00%).

### Intervention outcomes

**FIGURE 2:**
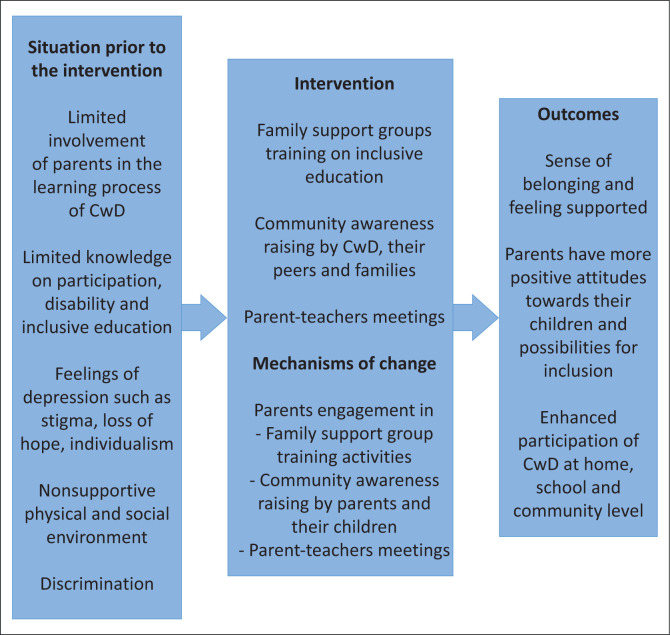
Diagrammatic representation of the parents’ intervention outcomes.

The summary results of the parents’ component of the *obuntu bulamu* intervention are shown in Figure 2. The thematic content analysis identified three recurrent themes from the participants’ narratives, which included: (1) belonging, (2) changing attitudes and (3) participation at the home, school and community level. In line with the intervention framework, these themes exhibited potential to improve inclusion of CwD at different levels. Under each theme, the different aspects, mechanisms of change and their outcomes are described.

### Acceptability

Parents generally said they enjoyed participating in the intervention activities. They expressed their gratitude for being included in the intervention design and evaluation of the study and appreciated meeting not only with the study team but also the teachers and other stakeholders during the consultative and validation meetings.

Attendance at the three training sessions was high, with 62 out of 64 parents attending the first, 62 out of 64 the second and 60 out of 64 parents attending the third training sessions. The training sessions were held at different schools participating in the study and typically lasted a full day. Breakfast, lunch and transport were provided. Parents explained that the proximity and familiarity of the training locations encouraged them to participate, as they was easy to reach and access and the timing allowed them to attend to their other daily tasks in the area as well. One of the parents during the FGD mentioned, ‘I can first do some work before I come because the place is near’.

The sessions also provided the opportunity to discuss and present infrastructural issues as a group within their children’s school and create linkages between the parents and teachers of the school.

The focal parents’ meetings for each term were held at the child’s school, with 30 out of 33 parents and teachers of CwD in the study (six meetings in total over a period of two years). The meeting sessions were always organised in the morning hours and lasted for 45 min – 60 min. According to the participants, this was convenient for them as it allowed them time to go back to their daily business. However, two participants reported to have missed out on sessions because the schedules clashed with their work.

Some participants initially thought that the focal parents could go ahead with the school meetings and represent the parents when discussing the child’s progress with the teachers. During follow-up visits, the focal parents explained to parents that their direct involvement during the school meetings was very important, as much as the focal parents were present to provide support. The focal parents visited all parents of CwD at their homes as well and also discussed inclusion in the home. During unannounced home visits, focal parents observed the different activities they would find a child engaged in, people who visited the home and the physical environment to facilitate accessibility discussions or interventions; they would also talk to the neighbours (with consent from the intervention participants) in case social inclusion barriers were identified. All parents completed the study, and there were no refusals or withdrawals.

### Belonging and feeling supported

Parents appreciated group-based activities, mentioning that the group experience was the most inspiring part of the intervention. They explained that at baseline, they used to think they were alone, had children with the worse conditions possible and had experienced the worst.

They mentioned that meeting with each other built their confidence and hope, increased a sense of belonging and reduced stigma. Participants in the parents’ FGD reported, ‘I keep learning from my fellow parents each time I come’, and ‘[*t*]his is a well-experienced group. Their stories have helped me’; another participant remarked that ‘[*l*]earning together took away my stress. Some of the mothers were very strong and would make you laugh’.

Both parents with and without CwD were very glad to meet with other parents to share their experiences and feel supported. For example, one of the FGD participants said, ‘having other parents with children with no disabilities participate with us was the best thing. We have got a new family. We no longer feel lonely like we used to’.

### Parents’ changing attitudes towards children with disabilities

At baseline, the majority of parents of CwD gave the impression that their children were ‘disabled’ and so had little they could do for school, home and community activities. Some felt very protective, not wanting their children to do homework, thinking it was a source of stress to them, whilst others had turned their CwD into ‘small queens’ (term used by parents to refer to someone who waits for others to work for them), hence paying no attention to fostering self-care. Similarly, parents might choose to keep their children at home because they believed they were not able to take care of themselves. Parents might feel protective and wish to prevent their children from having negative and harsh experiences, an attitude that most likely stemmed from a belief in the diminished aptitude of CwD. Persons with disabilities are often not valued by society. A number of parents described their child by their disability, for example, ‘the one with an impairment’ or ‘the one who cannot walk’, hence creating a barrier to their children’s inclusion. The majority of peer parents had not interacted with CwD before, they would say CwD were non-performers and they would avoid them or tell their children not to play with them.

At endline, however, the majority of parents described having a more positive attitude about the abilities of their children and the possibilities that could be created to include CwD at the family, school and community level. Parents started using different terminologies to refer to their CwD. Instead of referring to their child as *omulema* (the lame one) they would now say *omwana wange* (my child).

During some of the training sessions, persons with disabilities were invited to speak as peer role models. This was appreciated by parents, as they explained that it changed their attitude towards their children’s potential and future and gave them hope. One parent mentioned during an FGD that seeing trainers with disabilities ‘encouraged me so much’.

Some peer parents had attested at baseline that they were not comfortable having their children associate with CwD, as they used to worry about the causes of disability and were sceptical about CwD’s general potential, but they changed their story at endline. The peer parents explained that as a result of training and their coming together to interact with CwD and their parents, they approved of and even supported their children’s relationships with CwD:

‘I used to scold my son for spending much time with a child with disability, and he could always tell me that the boy is his best friend, that he has to support him. I didn’t know anything about this study; the first time I was invited for the training I was surprised because I do not have a child with disability […] What was taught helped me a lot to understand disability and those living with it. I never used to care for children with disabilities, but after the training, I developed love for these children that I wish all parents can get this kind of training, as it does not only benefit those having children with disabilities but also those without.’ (parent, son is 11 years old, peer child)

Parents said that their attitudes were important in changing children’s behaviour and that they had a role in teaching their children about inclusion. Peer parents in the FGD commented that ‘we parents should talk to our children on how to treat children with disabilities […] They should love and support children with disabilities’.

### Increased child participation at home, in school and the community

#### Home participation

Before the intervention, some parents mentioned that they used to ‘overprotect’ children by not allowing them to participate in any household activities because they were ‘disabled’. However, at endline, both parents of CwD and their peers explained that they were giving their children more roles and responsibilities such as cleaning around the home, washing personal clothes, preparing meals and entrusting them with money to do household shopping, as a result of trainings on child participation:

‘As parents, we had a tendency of not involving our children in day-to-day activities, and modifying our homes was not always a priority, but now I know the importance … I started encouraging her to participate with others in doing household chores, and she is happy.’ (parent, daughter is 8 years old, with disability)

Parents mentioned spending more time with their children, feeding them better or asking their siblings to play with them after the training sessions. Parents explained they had made learning or play materials from local materials together with their children after one of the sessions and were excited about their children’s creativity and ability. Parents felt that these changes could be attributed to the skills they acquired in the training and meetings.

#### School participation

Parents explained that they observed a positive change in school participation of CwD over the course of the study:

‘I have seen many good things since the onset of the study. Before it came, children with disabilities in regular schools were discriminated [*against*], but the study has trained teachers […] and have identified and empowered friends of the children and stopped discrimination […] The teachers and children are all informed, children with disabilities are treated like everyone else and are supported whenever there is need.’ (parent, daughter is 14 years old, with disability)

Parents’ endline reports showed increased involvement in their child’s learning process at school. They appreciated their new advocacy roles and awareness on the influence they had towards promoting inclusion:

‘Before our children had got their toilet, we discussed with my fellow parents, and we told them [*the school*] that we need a separate toilet for our children [*accessible to children with physical disabilities*] because the toilets were in a bad condition. The school listened and we got that toilet in our school, and we thank you so much for guiding us.’ (parent, daughter is 8 years old, with disability, FDG2)

In addition, parents expressed gratitude over the opportunity for the schools to discuss the child’s progress and concerns to ensure their participation during and outside class. Parents mentioned training as an eye opener to redefining of their roles to include checking on the child’s progress (how the child has been involved) at school and emphasising the value of education for CwD. They said they had increased their involvement in their children’s education and now visited their child’s school to meet with teachers more often to ensure their child is supported:

‘I am grateful because I was about to stop my son from going to school, as I did not know how to support him. I was paying school fees, but he wasn’t picking [*up*] anything from class, nor was he being promoted […] I used to think teachers were not doing their job, but since the start of this study, my understanding completely changed. The teachers now understand how to help him, and he has developed a love for school.’ (parent, son is 7 years old, with disability)

Parents of CwD also mentioned that they felt included in the educational planning process and that there was intensive cooperation. They eventually felt happy that their children were now more included in extracurricular activities in school such as music, dance and sports:

‘When my boy was in Primary 1, teachers used to feel sorry for him that they couldn’t let him do anything. He has a talent in dancing that as a parent I also know but he was never given a chance to join music as they thought he couldn’t manage, and I had also not taken an initiative to explain to them his potential. But after the training that we got on communicating the learning needs to teachers, I discussed with the teachers who too were positive, and my boy is involved in everything. I attend the school speech days knowing I will at least see my boy participating as I keep encouraging him.’ (parent, son is 10 years old, with a disability)

Focal parents and peer parents identified responsibilities and roles they assigned themselves in schools and communities based on the training and support they received. Parents explained that they wanted to create awareness about school inclusion in their communities:

‘What I have to do is marketing it to other parents in my area, I go on talking to them about the school and telling […] parents with children with a disability to take them to the school and not just leave them home.’ (parent, son is 12 years old, with disability)

#### Community participation

Primary care givers reported more support from their neighbours in the involvement of their CwD in community activities. The positive change was attributed to the peer-to-peer approach that encouraged parents of CwD and their peers to come together and get involved in similar activities. This kind of behaviour also fostered the same practice of children coming together and getting involved in similar activities, regardless of their abilities:

‘Sometimes when she [*child with disability*] has not gone to school, I leave her at home with the neighbours with some money to buy her what to eat; they also help me and give her tea, and by the time I come back, I find when she is fine playing with her friends like any other child.’ (parent, daughter is 12 years old, with disability, FGD1)

‘During birthdays, we would not invite others; neither would they invite us, but ever since we got to know his peer, we invite him and he comes along with others without disabilities.’ (parent, son is 10 years old, with disability, FDG2)

Parents felt a great improvement in social relationships in their communities. For example, one of the male parents explained that he did not know there was a child with a disability in the area where they resided. Bringing them together created a platform for interaction, and they can now support each other through play and company:

‘I didn’t know we were neighbours and had a common challenge. We are now free with one another, and our children are allowed to go to my friend’s home to play because I now know our son will be safe.’ (parent, son is 10 years old, with a disability)

Participants described that the intervention allowed free interaction within communities, as it enabled parents of CwD to freely talk about disability with their peer parents, teachers and other community members, addressing and responding to the diverse needs of CwD. Parents described the experience of working together as motivating and encouraging, as it promoted social interaction amongst parents, CwD and their peers:

‘My neighbours used to not to allow their children to play with our child, and her siblings too would leave her behind as they went to the neighbourhood to play. It used to hurt me, but ever since the team visited us and talked to them, each one care about her. They even collect some mangoes and bring for her. Neighbour’s children are now coming home, and they are her friends.’ (parent, daughter is 12 years old, with disability, FGD1)

Overall, parents felt that the *obuntu bulamu* intervention was an enjoyable and culturally appropriate intervention which can change attitudes towards CwD. Parents felt supported by the intervention and felt it improved belonging, participation and inclusion.

## Discussion

The *obuntu bulamu* peer-to-peer support intervention promotes a sense of belonging, togetherness and inclusion through peer-to-peer support (Bannink Mbazzi et al. 2020). In this study, the intervention was positively evaluated by parents of CwD and peer parents. The intervention enhanced a sense of togetherness and belonging, changed attitudes and practices and improved participation and inclusion of CwD at home, in school and in communities. The study’s findings supported the hypothesis that the peer-to-peer approach potentially supports participation in daily living activities and home interactions between household members and CwD, as well as inclusion in community activities and participation of CwD and their families in research.

The findings from the literature reviewed suggest that social difference, identity, power, local context and communal cultural values should be considered when studying inclusion and inclusive schooling. At baseline, parents reported the practices of isolating their children, naming them by their disability and considering participation in their learning process to be a waste of time. Such negative attitudes and practices are a key barrier to inclusion (Afolabi 2014; Afolabi, Mukhopadhyay & Nenty 2013). Parents’ attitudes towards a disability inclusion programme, including the implementation of an inclusive education, are important to promote inclusion (Paseka & Schwab 2020). Further assessment of parents’ attitudes and participation in inclusive education programmes should therefore be given high priority, according to a recommendation by Paseka and Schwab (2020).

Previous studies have corroborated this study’s findings and shown that increased parental involvement and support has a positive outcome on a child’s education and behaviour, and Newman (2000) observed that this sensitive support promotes the child’s continued engagement in learning activities. Parents in our study were enthusiastic about the parent awareness sessions and engagement in school meetings, reporting that they helped them understand and support their children better.

Similarly, attitude change amongst parents is critical in promoting belonging and togetherness, an area that has been recommended by scholars in the promotion of inclusion (Venkatakrishnashastry & Vranda 2012). One of the key themes in our study was the benefit of receiving peer-to-peer support. Parents in our study reported that training and group-based activities created a sense of belonging, hence feeling supported. Several researchers have highlighted how disability is a source of stress to parents (Weiss, Sullivan & Diamond 2003) and have emphasised the importance of parental involvement in disability-inclusive interventions to improve their child’s outcomes (Blue-Banning et al. 2004). In our intervention, parents described a beneficial outcome for them and their child, which is an important part of the *obuntu bulamu* approach, which emphasises collective belonging and responsibility towards each other rather than individual child outcomes alone.

Harris et al. (2015) argued that peer-to-peer support improves community participation. Similarly, Lloyd, Tse and Deane (2006) added that ongoing and good quality support is needed to promote social integration of persons with disabilities and their members in the community. Parents in our study appreciated the peer-to-peer support received from their neighbours and community and the roles each person could play in supporting one another to collectively achieve a more inclusive environment for all children. However, female parents reported that the limited engagement of their male counterparts was limiting their full support as female parents at both community and family level. It is clear from earlier research that father involvement has enormous implications for men on their own path of adult development, for their wives and partners in the co-parenting relationship and, most importantly, for their children in terms of social, emotional and cognitive development (Allen & Daly 2002; Siu et al. 2017). A larger study is now being planned to increase men’s involvement by including fathers’ training, meetings and their engagement as role models.

In conclusion, the *obuntu bulamu* intervention demonstrated that it is possible to significantly change parental attitudes towards disability inclusion and increase participation and inclusion of CwD in homes, schools and communities.

## Summary of major findings and shortcomings

This study describes parent participation and evaluation of a Ugandan intervention for children, parents and teachers which aims to improve the participation and inclusion of CwD. Parents perceived the programme as an acceptable, culturally appropriate and supportive intervention which can potentially enhance participation and inclusion of CwD at home, in schools and communities. The main shortcomings were the relatively small sample size (*n* = 64) in Central Uganda only. The intervention needs to be further tested in a larger study population to be able to generalise findings (a trial was underway at the time of writing this manuscript).
